# An Enhanced Decoding Algorithm for Coded Compressed Sensing with Applications to Unsourced Random Access

**DOI:** 10.3390/s22020676

**Published:** 2022-01-16

**Authors:** Vamsi K. Amalladinne, Jamison R. Ebert, Jean-Francois Chamberland, Krishna R. Narayanan

**Affiliations:** 1 Qualcomm Technologies, Inc., San Diego, CA 92121, USA; vamsia@qti.qualcomm.com; 2 Department of Electrical and Computer Engineering, Texas A&M University, College Station, TX 77843, USA; jrebert@tamu.edu (J.R.E.); krn@tamu.edu (K.R.N.)

**Keywords:** concatenated codes, successive cancellation list decoding, coded compressed sensing, unsourced random access

## Abstract

Unsourced random access (URA) has emerged as a pragmatic framework for next-generation distributed sensor networks. Within URA, concatenated coding structures are often employed to ensure that the central base station can accurately recover the set of sent codewords during a given transmission period. Many URA algorithms employ independent inner and outer decoders, which can help reduce computational complexity at the expense of a decay in performance. In this article, an enhanced decoding algorithm is presented for a concatenated coding structure consisting of a wide range of inner codes and an outer tree-based code. It is shown that this algorithmic enhancement has the potential to simultaneously improve error performance and decrease the computational complexity of the decoder. This enhanced decoding algorithm is applied to two existing URA algorithms, and the performance benefits of the algorithm are characterized. Findings are supported by numerical simulations.

## 1. Introduction

Massive machine-type communication (mMTC) is a rapidly growing class of wireless communications that aim to connect tens of billions of unattended devices to wireless networks. One significant application of mMTC is that of distributed sensing, which consists of a large number of wireless sensors that gather data over time and transmit their data to a central server. This server then interprets the received data to produce useful information and/or make executive decisions. When combined with recent advances in machine learning, such networks are expected to open a vast realm of economic and academic opportunities. However, the large population of unattended devices within these networks threatens to overwhelm existing wireless communication infrastructures by dramatically increasing the number of network connections; it is expected that the number of machines connected to wireless networks will soon exceed the population of the planet by at least an order of magnitude. Additionally, the sporadic and bursty nature of sensor transmissions makes them highly inefficient under current estimation/enrollment/scheduling procedures typical of cellular networks [1,2]. The combination of these challenges necessitates the design of novel physical and medium access control (MAC) layer protocols to efficiently handle the demands of these wireless devices.

One recently proposed paradigm for efficiently handling the demands of unattended devices is that of unsourced random access (URA), as described by Polyanskiy in 2017 [3]. URA captures many of the nuances of IoT devices by considering a network with an exceedingly large number of uncoordinated devices, of which only a small percentage is active at any given point in time. When a device/user is active, it encodes its short message using a common codebook and then transmits its codeword over a regularly scheduled time slot, as facilitated by a beacon. Furthermore, the power available to each user is strictly limited and assumed to be uniform across devices. The use of a common codebook is characteristic of URA and has two important implications: First, the network does not need to maintain a dictionary of active devices and their unique codebook information; second, the receiver does not know which node transmitted a given message unless the message itself contains a unique identifier. The receiver is then tasked with recovering an unordered list of transmitted messages sent during each time slot by the collection of active devices. The performance of URA schemes is evaluated with respect to the per-user probability of error (PUPE), which is the probability that a user’s message is not present in the receiver’s final list of decoded messages; this criterion is formally defined in (3). In [3], Polyanskiy provides finite block length achievability bounds for short block lengths typical of URA applications using random Gaussian coding and maximum likelihood (ML) decoding. However, these bounds are derived in the absence of complexity constraints and, thus, are impractical for deployment in real-world networks. Over the past few years, several alternative URA schemes have been proposed that offer tractable complexity with minor degradation in performance. For example, [4,5,6,7,8,9] exploit connections between URA and compressed sensing (CS) to reduce decoding complexity. In [10,11,12,13,14,15,16,17,18,19], schemes are presented that seek to separate user’s signals and recover them using single-user coding techniques. These results are extended to multiple-input multiple-output (MIMO) scenarios in [20,21,22,23,24,25].

All of the aforementioned URA schemes employ concatenated channel codes to recover messages sent by the collection of active users at the receiver. We note that the term *channel code* is used broadly such that it includes spreading sequences and certain signal dictionaries such as those commonly used for CS. Although it is conceptually simpler to decode inner and outer codes separately, it is a well-known fact within coding theory that dynamically sharing information between the inner and outer decoders will often improve the performance of the system [26,27]. In this paper, we present a novel framework for sharing information between a wide class of inner codes and a tree-based outer code. This approach significantly improves PUPE performance and reduces the computational complexity of the scheme. Specifically, our main contributions are as follows:

1 A general system model consisting of a wide class of inner codes and an outer tree code is developed. An enhanced decoding algorithm is presented whereby the outer tree code may guide the convergence of the inner code by restricting the search space of the inner decoder to parity consistent paths.
2 The coded compressed sensing (CCS) scheme of Amalladinne et al. in [5] is considered under this model. With the enhanced decoding algorithm, the $E_{b}/N_{0}$ required to achieve a fixed PUPE is reduced by nearly 1 dB when the number of users is low and the number of columns in the sensing matrix is reduced by over 99% by the last slot, thus significantly reducing decoding complexity.
3 The CCS for massive MIMO scheme of Fengler et al. in [21] is considered under this model. With the enhanced decoding algorithm, the number of antennas required to achieve a fixed PUPE is reduced by 23% in certain regimes, and the decoding runtime is reduced by 70–90%.

## 2. System Model

Consider a URA scenario consisting of *K*-active devices, which are referred to by a fixed but arbitrary label j∈[K]. Each of these users wishes to simultaneously transmit a *B*-bit message $w_{j}$ to a central base station over a Gaussian multiple access channel (GMAC) using a concatenated code consisting of an inner code C and an outer tree code T. This inner code C has the crucial property that, given a linear combination of K≤δ codewords, the constituent information messages may be individually recovered with high probability. Furthermore, we assume that the probability that any two active users’ messages that are identical is low, i.e., $\mathrm{Pr}(w_{i}=w_{j})<\epsilon$ whenever i≠j.

We consider a scenario where it is either computationally intractable to inner encode/decode the entire message simultaneously or it is otherwise impractical to transmit the entire inner codeword at once. Thus, each user must divide its information message into fragments and inner encode/decode each fragment individually. In order to ensure that the message can be reconstructed from its fragments at the receiver, the information fragments are first connected together by using the outer tree-based code T and then inner encoded using code C. The resulting signal is transmitted over the channel. We elaborate on this process below.

**Figure 1:**
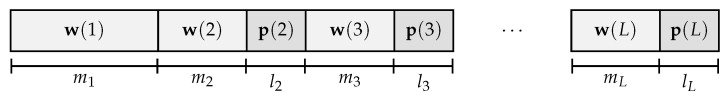
This figure illustrates the structure of a user’s outer encoded message, denoted by v. Fragment *ℓ* consists of the concatenation of information bits, denoted by w(ℓ), and parity bits, denoted by p(ℓ).

Each message $w_{j}$ is broken into *L* fragments where fragment *ℓ* has length $m_{\ell }$ and $\sum_{\ell \in [L]}m_{\ell }=B$. Notationally, $w_{j}$ is represented as the concatenation of fragments by $w_{j}=w_{j}\left(1\right)w_{j}\left(2\right)\cdots w_{j}\left(L\right)$. The fragments are outer-encoded together by adding parity bits to the end of each fragment, with the exception of the first fragment. This is accomplished by taking random linear combinations of the information bits contained in previous sections. The parity bits appended to the end of section *ℓ* are denoted by $p_{j}\left(\ell \right)$, and they collectively have length $l_{\ell }$. This outer-encoded vector is denoted by $v_{j}$, where $v_{j}\left(\ell \right)=w_{j}\left(\ell \right)p_{j}\left(\ell \right)$. The vector $v_{j}$ now assumes the form shown in Figure 1.

At this point, it may be instructive to explain our preference for the tree code over possible alternatives such as LDPC codes, polar codes, and BCH codes. The main purpose of the tree code is disambiguation, as opposed to error correction. It offers a principled method to trade-off performance and complexity, as detailed in [5]. Furthermore, it provides optimal asymptotic performances, as shown in [6]. It remains unclear how the aforementioned alternative coding techniques would work in this current context. This situation acts as a strong motivation for the development of the tree code in [5] and for its adoption in [4,6,21] as well as within this manuscript.

After the outer-encoding process is complete, user *j* inner encodes each fragment $v_{j}\left(\ell \right)$ individually using C and concatenates the encoded fragments to form signal $x_{j}$. Each user then simultaneously transmits its signal to the base station over a GMAC. The received signal at the base station assumes the following form:

(1) $$y=\sum_{j\in [K]}dx_{j}+z$$

where z is a vector of Gaussian noise with independent standard normal components, and *d* accounts for the transmit power.

**Figure 2:**
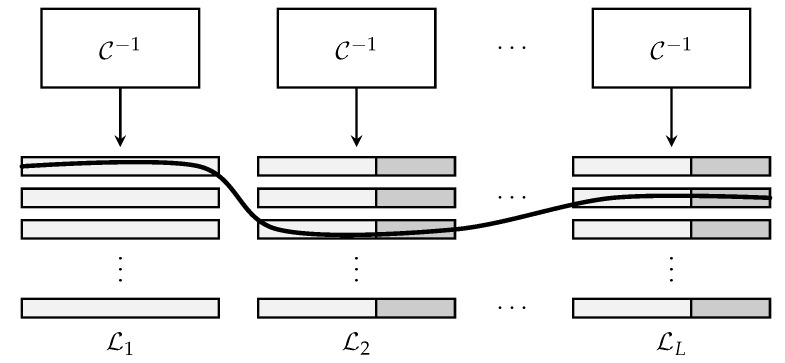
This figure illustrates the operation of the tree decoder. The inner decoder $C^{-1}$ produces *L* lists of *K* messages each. The outer tree decoder then finds parity-consistent paths across lists to extract valid messages.

Recall that the receiver is tasked with producing an unordered list of all transmitted messages. A naive method to perform this is to have inner and outer decoders operate independently of each other. That is, the inner decoder is first run independently on each of the *L* sections in y. Since C has the property that, given a linear combination of its codewords, the constituent input signals may be recovered with high probability, the aggregate signal in every slot can be expanded into a list of *K* encoded fragments $\left\{{\hat{v}}_{j}\left(\ell \right):j\in \left[K\right]\right\}$. It may be helpful to remind the reader that ${\hat{v}}_{j}\left(\ell \right)$ does not necessarily correspond to the message sent by user *j* because the receiver has no way of connecting a received message to an active user within URA. Therefore, at this stage, the receiver has *L* unordered lists $L_{1},L_{2},\cdots ,L_{L}$, each with *K* outer-encoded fragments. From these lists, the receiver wishes to recover *K* messages sent by the active devices during the frame. This is performed by running the tree decoder on the *L* lists to find parity-consistent paths across lists. Specifically, the tree decoder first selects a root fragment from list $L_{1}$ and computes the corresponding parity section p(2). The tree decoder then branches out to all fragments in list $L_{2}$, for which its parity sections match p(2); each match creates a parity-consistent partial path. This process repeats until the last list $L_{L}$ is processed. At this point, if there is a single path from $L_{1}$ to $L_{L}$, the message created by that path is deemed valid and stored for further processing. On the other hand, if there are multiple parity-consistent paths from a given root fragment or no parity consistent paths from a given root fragment, a decoding failure is declared. Figure 2 illustrates this scheme.

While intuitive, this strategy is sub-optimal because information is not being shared by the inner and outer decoders. If the inner and outer decoders were to operate concurrently, the output of the outer decoder could be used to reduce the search space of the inner decoder, thus guiding the convergence of the inner decoder to a parity-consistent solution. This would also reduce the search space of the inner code, thus providing an avenue for reducing decoding complexity [28,29]. Explicitly, assume that immediately after the inner decoder produces list $L_{\ell }$, the outer decoder finds all parity-consistent partial paths from the root node to stage *ℓ*. Each of these *R* parity-consistent partial paths has an associated parity section $p_{r}\left(\ell +1\right)$. Furthermore, it is known that only those fragments in $L_{\ell +1}$ that contain one of the $\left\{p_{r}\left(\ell +1\right):r\in \left[R\right]\right\}$ admissible parity sections may be part of *K*-transmitted messages. Thus, when producing $L_{\ell +1}$, the search space of the inner decoder may be reduced drastically to only the subset for which fragments contain an admissible parity section $p_{r}\left(\ell +1\right)$.

This algorithmic enhancement is a key contribution; it has the potential to simultaneously reduce decoding complexity and improve PUPE performance. Still, a precise characterization of the benefits of this enhanced algorithm depends on the inner code chosen. We now consider two situations in which this algorithm may be applied: coded compressed sensing (CCS) [5] and CCS for massive MIMO [21]. For each of the considered schemes, complexity reduction and performance improvements are quantified. We emphasize that this algorithmic enhancement is applicable to other scenarios beyond those considered in this paper. One such example is the CHIRRUP scheme presented by Calderbank and Thompson in [4]. This latter scheme uses an efficient CS solver based on a second order Reed-Muller code concatenated with a tree outer code as the foundation for a divide-and-conquer approach. The decoding process is performed by using a depth-first search within inner blocks, followed by stitching. The depth-first search of later blocks could potentially be informed by the type of search space pruning described herein. Details are omitted due to space considerations.

## 3. Case Study 1: Coded Compressed Sensing

CCS has emerged as a practical scheme for URA that offers good performance with low complexity [5,6,7,8,9]. We note briefly that some recent variants of CCS that employ an LDPC outer code [7,8,9] are not compatible with the model presented in this paper. Thus, we focus on the original version of the algorithm presented by Amalladinne et al. in [5]. At its core, CCS seeks to exploit a connection between URA and compressed sensing (CS). This connection may be understood by transforming a *B*-bit message w into a length $2^{B}$ index vector m, where the single non-zero entry is a one at location ${\left[w\right]}_{2}$. The notation ${\left[w\right]}_{2}$ denotes binary message w interpreted as a radix-10 integer. This bijection between w and m is denoted by f(·). The vector m may then be compressed into signal x using sensing matrix A, where A is an appropriately chosen CS matrix. We note that there is some flexibility in the selection of A: Gaussian, Rademacher, and sub-sampled Hadamard/DFT matrices are suitable choices. Additionally, LDPC-based or other structured sensing matrices may be employed instead. Regardless of the choice of A, the columns of A must be normalized such that $E[A_{:,i}]=1$ for every *i* in order to satisfy URA power constraints.

Each device then transmits its signal over the noisy multiple access channel, which naturally adds the sent signals together. At the receiver, the original signals may be recovered from y using standard CS recovery techniques such as non-negative least-squares (NNLS) or least absolute shrinkage and selection operator (LASSO). However, for messages of even modest lengths, the size of x is too large for standard CS solvers to handle. In order to circumvent this challenge, a divide and conquer approach can be employed.

In CCS, inner code C consists of the CS encoder, and the outer tree code T is identical to that presented in Section 2. Note that there is an additional step between T and C: The outer-encoded message v is transformed into the inner code input m via the bijection f(·) described above. Furthermore, we emphasize that C has the property that, given a linear combination of K≤δ codewords, the corresponding set of *K* one-sparse constituent inputs may be recovered with high probability. This, combined with the assumption that $\mathrm{Pr}(w_{i}=w_{j})<\epsilon$ for i≠j, makes CCS an eligible candidate for the enhanced decoding algorithm described previously. We review below CCS encoding and decoding operations.

### 3.1. CCS Encoding

When user *j* wishes to transmit a message to the central base station, it encodes its message in the following manner. First, it breaks its *B*-bit message into *L* fragments and outer-encodes the *L* fragments using the tree code described in Section 2; this yields outer codeword $v_{j}$. Recall that fragment *ℓ* has $m_{\ell }$ information bits and $l_{\ell }$ parity bits. We emphasize that $m_{\ell }+l_{\ell }=v_{\ell }$ is constant for all sections in CCS, but the ratio of $m_{\ell }$ to $l_{\ell }$ is subject to change. Fragment $v_{j}\left(\ell \right)$ is then converted into length $2^{m_{\ell }+l_{\ell }}$ index vector, denoted by $m_{j}\left(\ell \right)$, and compressed using sensing matrix A into vector $x_{j}\left(\ell \right)$. Within the next transmission frame, user *j* sends its encoded fragments across GMAC along with all other active users. At the base station, the received vector associated with slot *ℓ* assumes the following form:

(2) $$y\left(\ell \right)=\sum_{j\in [K]}dAm_{j}\left(\ell \right)+z\left(\ell \right)=dA\sum_{j\in [K]}m_{j}\left(\ell \right)+z\left(\ell \right)$$

where z(ℓ) is a vector of Gaussian noise with standard normal components, and *d* reflects the transmit power. This is a canonical form of a *K*-sparse compressed vector embedded in Gaussian noise.

### 3.2. CCS Decoding

CCS decoding begins by running a standard CS solver such as NNLS or LASSO on each section to produce *LK*-sparse vectors. The *K* indices in each of these *L* slots are converted back to binary representations using $f^{-1}\left(\cdot \right)$, and the tree decoder is run on resultant *L* lists to produce estimates of transmitted messages.

**Figure 3:**
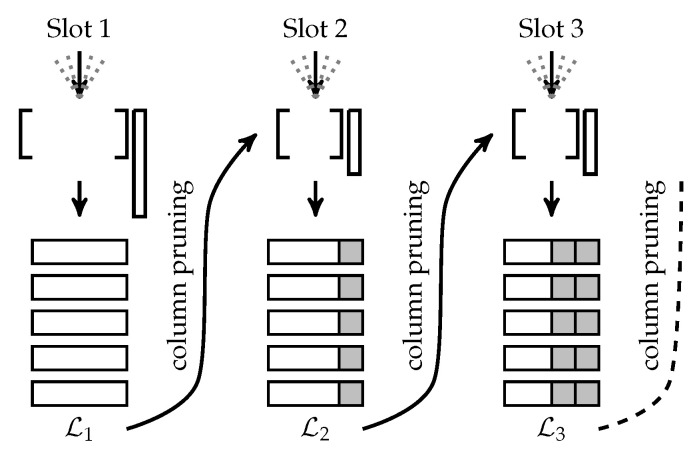
This figure illustrates the enhanced decoding algorithm applied to CCS. After recovering $L_{\ell }$, the sensing matrix A is pruned so that list $L_{\ell +1}$ only contains parity-consistent fragments.

This process may be improved by applying the proposed enhanced decoding algorithm, which proceeds as follows for CCS. The inner CS solver first recovers section 1, and then computes the set of possible parity patterns for section 2, denoted by $P_{2}$. The columns of A are then pruned dynamically to remove all columns associated with inadmissible parity patterns in section 2. This reduces the number of columns of A from $2^{m_{1}+l_{1}}$ to $2^{m_{1}}\left|P_{1}\right|$ [28]. Section 2 is then recovered, and the process repeats itself until section *L* has been decoded. At this point, valid paths through the *L* lists are identified, and the list of estimated transmitted messages is finalized. Figure 3 illustrates this process.

### 3.3. Results

As previously mentioned, the algorithmic enhancement presented in this article has the potential to improve both the performance and computational complexity of certain concatenated coding schemes. Before quantifying the gains obtained by applying this algorithmic enhancement to CCS, we define appropriate measures of performance and computational complexity. Being a URA scheme, the performance of CCS is evaluated with respect to the per-user probability of error (PUPE), which is given by the following:

(3) $$P_{e}=\frac{1}{K}\sum_{j\in [K]}\mathrm{Pr}\left(w_{j}\notin \hat{W}\left(y\right)\right)$$

where $\hat{W}\left(y\right)$ is the estimated list of transmitted messages, with at most *K* items. Since many different CS solvers with varying computational complexities may be employed within the CCS framework, the complexity reduction offered by the enhanced decoding algorithm is quantified by counting the number of columns removed from A.

The column pruning operation has at least four major implications on the performance and complexity of CCS. These implications are summarized below.

1 Many CS solvers rely on iterative methods or convex optimization solvers to recover x from y=Ax. Decreasing the width of A will result in a reduction in computational complexity, the exact size of which will depend on the CS solver employed.
2 When all message fragments have been correctly recovered for stages 1,2,⋯,ℓ, the matrix A is pruned in such a way that is perfectly consistent with the true signal. In this scenario, the search space for the CS solver is significantly reduced and the performance will improve.
3 When an erroneous message fragment has been incorrectly identified as a true message fragment by stage *ℓ*, the column pruning operation will guide the CS solver to a list of fragments that is more likely to contain additional erroneous fragments. This further propagates the error and helps erroneous paths stay alive longer.
4 When a true fragment is mistakenly removed from a CS list, its associated parity pattern may be discarded and disappear entirely. This results in the loss of a correct message and additional structured noise, which may decrease the PUPE performance of other valid messages.

**Figure 4:**
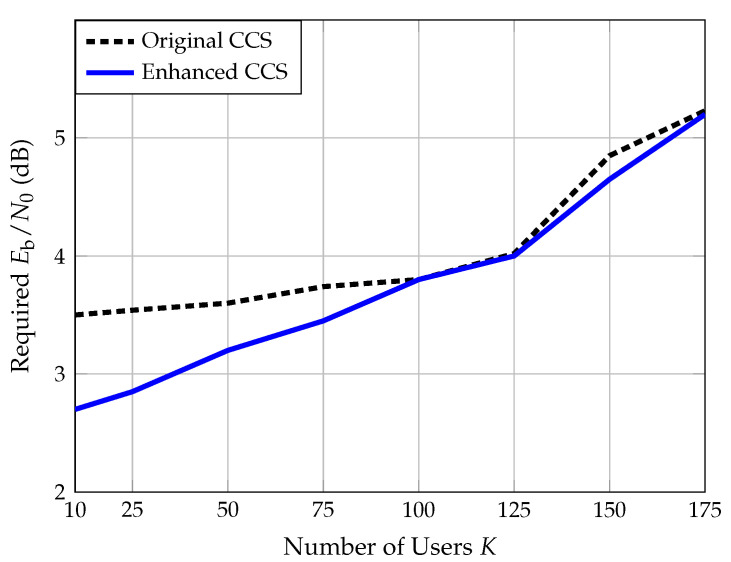
This figure shows the required $E_{b}/N_{0}$ to obtain a PUPE of 5% versus the number of active users.

Despite having positive and negative aspects, the net effect of the enhanced decoding algorithm on the system’s PUPE performance is positive, as illustrated in Figure 4. This figure was generated by simulating a CCS scenario with K∈[10:175] users, each of which wishes to transmit a B=75 bit message divided into L=11 stages over 22,517 channel uses. NNLS was used as the CS solver.

From Figure 4, we observe that the enhanced decoding algorithm reduces the required $E_{b}/N_{0}$ by nearly 1 dB for a low number of users. Furthermore, for the entire range of number of users considered, the enhanced algorithm is at least as good as the original algorithm and often much better.

By tracking the expected number of parity-consistent partial paths, it may be possible to compute the expected column reduction ratio at every stage. However, this is a daunting task, as explained in [5]. Instead, we estimate the expected column reduction ratio by applying the approximate analysis found in [5], which relies on the following simplifying assumptions:

- No two users have the exact same message fragments at any stage: $w_{i}\left(\ell \right)\neq w_{j}\left(\ell \right)$ whenever i≠j and for all ℓ∈[L].
- The inner CS decoder makes no mistakes in producing lists $L_{1},\cdots ,L_{L}$.

Under these assumptions and starting from a designated root node, the number of erroneous paths that survive stage *ℓ*, denoted $L_{\ell }$, is subject to the following recursion.

(4) $$\begin{array}{cc} E\left[L_{\ell }\right]\; & =E[E\left[L_{\ell }|L_{\ell -1}\right]]\\ \; & =E\left[\left(\left(L_{\ell -1}+1\right)K-1\right)2^{-l_{\ell }}\right]\\ \; & =2^{-l_{\ell }}KE\left[L_{\ell -1}\right]+2^{-l_{\ell }}\left(K-1\right). \end{array}$$

Using initial condition $E[L_{1}]=0$, we obtain the following expected value.

(5) $$E\left[L_{\ell }\right]=\sum_{q=2}^{\ell }\left(K^{\ell -q}\left(K-1\right)\prod_{k=q}^{\ell }2^{-l_{k}}\right).$$

When matrix A is pruned dynamically, then *K* copies of the tree decoder run in parallel and, as such, the expected number of parity-consistent partial paths at stage *ℓ* can be expressed as follows.

$$P_{\ell }=K\left(1+E\left[L_{\ell }\right]\right).$$

Under the further simplifying assumptions that all parity patterns are independent and $P_{j}$ concentrates around its mean, we can approximate the number of admissible parity patterns. The probability that a particular path maps to a specific parity pattern is $2^{-l_{\ell }}$; hence, the probability that this pattern is not selected by any path become ${\left(1-2^{-l_{\ell }}\right)}^{P_{\ell }}$. Taking the complement of this event and multiplying by the number of parity patterns, we obtain an approximate expression for the mean number of admissible patterns.

(6) $$|P_{\ell }|\approx 2^{l_{\ell }}\left(1-{\left(1-2^{-l_{\ell }}\right)}^{P_{\ell }}\right).$$

Thus, the expected column reduction ratio at slot *ℓ*, denoted $E[R_{\ell }]$, is provided by the following.

(7) $$E\left[R_{\ell }\right]=1-{\left(1-2^{-l_{\ell }}\right)}^{P_{\ell }}.$$

**Figure 5:**
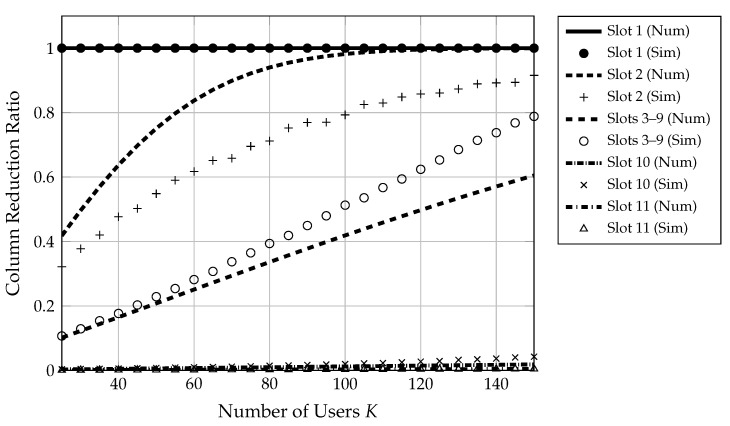
This figure illustrates the column reduction ratio provided by the enhanced decoding algorithm for each stage of the outer code and a varying number of users. Lines represent numerical results, and markers represent simulated results. Clearly, the size of the sensing matrix may be drastically reduced.

Figure 5 shows the estimated versus simulated column reduction ratio across stages. Overall, the number of columns in A can be reduced drastically for some stages, thus significantly lowering the complexity of the decoding algorithm.

## 4. Case Study 2: Coded Compressed Sensing for Massive MIMO

A natural extension of the single-input single-output (SISO) version of CCS proposed in [5] is a version of CCS where the base station utilizes M≫1 receive antennas. In this scenario, we assume that the receive antennas are sufficiently separated to ensure negligible spatial correlation across channels. Furthermore, we adopt a block fading model where the channel remains fixed for a coherence period of *n* channel uses and all coherence blocks are assumed to be completely independent, as in [20]. Each active user transmits its message over *L* coherence blocks, with one coherence block corresponding to each of the *L* sections described above; thus, the total number of channel uses is N=nL. As in SISO CCS, the receiver is tasked with producing an estimated list of the messages transmitted by the collection of active users during a given time instant. In addition to observing the received signal, the base station has knowledge of the total number of active users, the codes used for encoding messages, and the second-order statistics of MIMO channels. We note that channel state information (CSI) is not fully known. Thus, the decoding algorithm can be characterized as non-coherent [29]. The scheme we consider in this study was first presented by Fengler et al. in [21].

### 4.1. MIMO Encoding

The encoding process for CCS with massive MIMO is analogous to the encoding process for CCS; for a thorough description of this process, please refer to Section 3. However, the signal received by the base station will have a different structure as the base station features *M* receive antennas. Let x(t,ℓ) denote the *t*th symbol in block *ℓ* of vector x. Then, the signal observed at the base station is of the following form:

(8) $$y\left(t,\ell \right)=\sum_{j\in [K]}x_{j}\left(t,\ell \right)h_{j}\left(\ell \right)+z\left(t,\ell \right)\;t\in \left[n\right],\;\ell \in \left[L\right]$$

where z(t,ℓ) is circularly symmetric complex white Gaussian noise with zero mean and variance $N_{0}/2$ per dimension and $h_{j}\left(\ell \right)∼\mathrm{CN}\left(0,I_{M}\right)$ is a vector of small-scale fading coefficients representing the channel between user *j* and the base station’s *M* antennas.

### 4.2. MIMO Decoding

Recall that a URA receiver is tasked with producing an unordered list of the messages transmitted by the collection of active devices. To perform this, the receiver must first identify the list of fragments transmitted during each of the *L* coherence blocks and then extract the transmitted messages by finding parity consistent paths across lists. The receiver architecture presented in [21] features a concatenated code, where the inner code C is decoded using a covariance-based activity detection (AD) algorithm and the outer tree code T is decoded in a manner identical to that presented in Section 2.

Recall that each active user transforms its outer-encoded message v into a 1-sparse index vector m. Let $\left\{i_{j}\left(\ell \right):j\in \left[K\right]\right\}$ denote the set of indices chosen by the active users during block *ℓ*. Then, the signal observed at the base station is of the following form:

(9) $$\begin{array}{cc} Y(\ell ) & =\sum_{j\in [K]}a_{i_{j}\left(\ell \right)}\left(\ell \right)h_{j}{\left(\ell \right)}^{⊺}+Z\left(\ell \right)\\ \; & =A(\ell )\Gamma (\ell )H(\ell )+Z(\ell ) \end{array}$$

where H(ℓ) has independent CN(0,1) entries, Z(ℓ) is an independent complex Gaussian noise, and Γ(ℓ) is a diagonal matrix that indicates which indices have been selected during block *ℓ*; that is, $\Gamma \left(\ell \right)=\mathrm{diag}(\gamma_{0}\left(\ell \right),\cdots ,\gamma_{2^{v_{\ell }}}\left(\ell \right))$ with the following.

(10) $$\gamma_{i}\left(\ell \right)=\left\{\begin{array}{cc} 1 & i\in \{i_{j}\left(\ell \right):j\in \left[K\right]\}\\ 0 & \mathrm{otherwise}. \end{array}\right.$$

**Figure 6:**
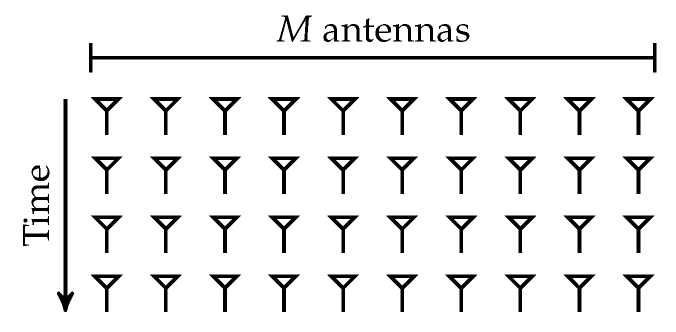
This figure illustrates the structure of Y(ℓ), where the rows correspond to time instants, and the columns correspond to receive antennas.

Finally, Y(ℓ) is an n×M matrix where the rows of Y(ℓ) correspond to various time instants and the columns of Y(ℓ) correspond to the different antennas present at the base station. Figure 6 illustrates this configuration.

Determining which fragments were sent during coherence block *ℓ* is equivalent to estimating Γ(ℓ). This process is referred to as activity detection and may be accomplished through covariance matching when the number of receive antenna is large. An iterative algorithm for estimating Γ(ℓ) was first proposed by Fengler in [21] and is summarized in Algorithm 1. After the collection of fragments transmitted in each of the *L* sub-blocks has been recovered by Algorithm 1, tree decoding is employed to disambiguate the collection of transmitted messages.
**Algorithm 1 **Activity Detection via Coordinate Descent1:**Inputs**: Sample covariance ${\hat{\Sigma }}_{Y(\ell )}=\frac{1}{M}Y\left(\ell \right)Y{\left(\ell \right)}^{H}$2:**Initialize**: $\Sigma_{\ell }=N_{0}I_{n}$, γ(ℓ)=03:**for**i=1,2,…**do**4:    **for** $k\in S_{\ell }$ **do**5:        Set $d^{*}=\frac{a_{k}{\left(\ell \right)}^{H}\Sigma_{\ell }^{-1}\left({\hat{\Sigma }}_{Y(\ell )}\Sigma_{\ell }^{-1}-I_{n}\right)a_{k}\left(\ell \right)}{{\left(a_{k}{\left(\ell \right)}^{H}\Sigma_{\ell }^{-1}a_{k}\left(\ell \right)\right)}^{2}}$6:        Update $\gamma_{k}\left(\ell \right)\leftarrow \max\left\{\gamma_{k}\left(\ell \right)+d^{*},0\right\}$7:        Update $\Sigma_{\ell }^{-1}\leftarrow \Sigma_{\ell }^{-1}-\frac{d^{*}\Sigma_{\ell }^{-1}a_{k}\left(\ell \right)a_{k}{\left(\ell \right)}^{H}\Sigma_{\ell }^{-1}}{1+d^{*}a_{k}{\left(\ell \right)}^{H}\Sigma_{\ell }^{-1}a_{k}\left(\ell \right)}$8:**Output**: Estimate γ(ℓ)

As before, it is possible to leverage the enhanced version of the tree decoding process, with its dynamic pruning, to improve performance and lower complexity. The application of the proposed algorithmic enhancement to the activity detection algorithm may be visualized in the following manner. Let $S_{\ell }$ denote the set of indices to perform coordinate descent over during coherence block *ℓ*; in its original formulation, $S_{\ell }=\left[2^{v_{\ell }}\right]$. After, list $L_{1}$ has been produced by the activity detection algorithm, and the tree decoder can compute the set of all admissible parity patterns $P_{2}$ for list $L_{2}$; then, A(2) may be pruned to only contain columns corresponding to messages with parity patterns in $P_{2}$. A similar strategy can be applied moving forward, yielding a reduced admissible set $P_{\ell }$ for parity patterns at stage *ℓ*. In turn, this reduces the index set $S_{\ell }$ to the following:

(11) $$S_{\ell }=\left\{{\left[w\left(\ell \right)p\left(\ell \right)\right]}_{2}:w\left(\ell \right)\in {\left\{0,1\right\}}^{m_{\ell }},p\left(\ell \right)\in P_{\ell }\right\}$$

which may be significantly smaller than $\left[2^{v_{\ell }}\right]$. This algorithmic refinement guides the activity detection algorithm to a parity-consistent solution and reduces the search space of the inner decoder, thus improving performance significantly [29].

### 4.3. Results

**Figure 7:**
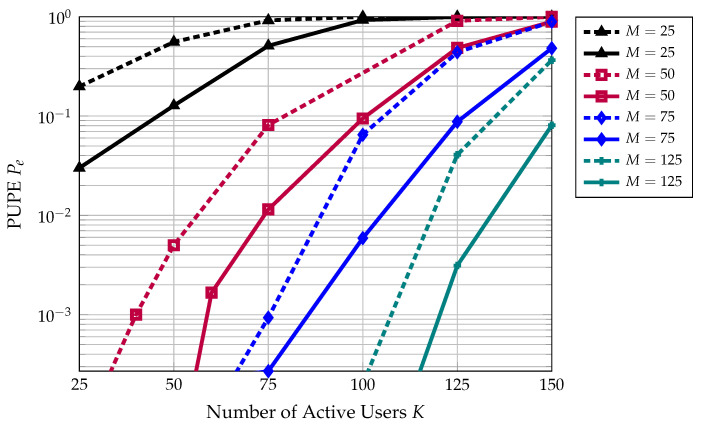
This figure illustrates the performance advantage of applying the enhanced decoding algorithm presented in this paper to CCS for massive MIMO. For a fixed number of antennas, the dashed line represents the original performance from [21], and the solid line represents the performance of the enhanced algorithm.

The simulation results presented in this section correspond to a scenario with K∈[25,150] active users and M∈[25,125] antennas at the base station. Each user encodes their 96-bit signal into L=32 blocks with 100 complex channel uses per block. The length of the outer-encoded block is $v_{\ell }=12$ for all ℓ∈[L], and a parity profile of $\left(l_{1},l_{2},\cdots ,l_{L}\right)=\left(0,9,9,\cdots ,9,12,12,12\right)$ is employed. The energy per bit $E_{b}/N_{0}$ is fixed at 0 dB, and the columns of A(ℓ) are chosen randomly from a sphere of radius $\sqrt{nP}$. These parameters are chosen to match [21]. Figure 7 shows the PUPE of this scheme for a range of active users and several different values of *M*. In this figure, the dashed lines represent the performance of the original algorithm and the solid lines represent the performance of the enhanced version with dynamic pruning.

**Figure 8:**
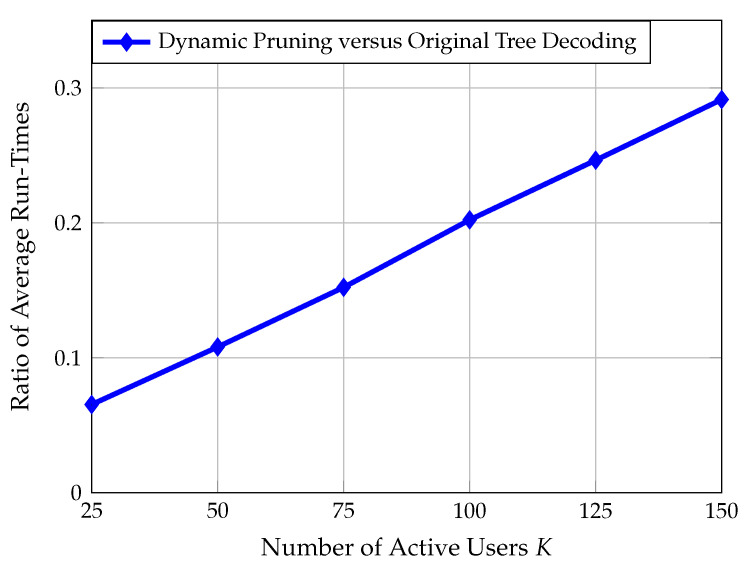
This figure plots the ratio of average runtimes between the enhanced decoding algorithm and the original CCS for massive MIMO scheme [21]. As observed above, dynamic pruning offers a significant reduction in computational complexity compared to standard tree decoding.

From Figure 7, we gather that the proposed algorithm reduces the PUPE for a fixed number of active users and a fixed number of antennas at the base station. Additionally, this algorithm may be used as a means to reduce the number of antennas required to achieve a target PUPE. For instance, when K=100, the enhanced algorithm allows for a 23% reduction in the number of antennas at the base station with no degradation in error performance. Figure 8 provides the ratio of average runtimes of the enhanced decoding algorithm versus the original decoding algorithm. The enhanced decoding algorithm also offers a significant reduction in computational complexity, especially for a low number of active users.

## 5. Conclusions

In this article, a framework for a concatenated code architecture consisting of a structured inner code and an outer tree code was presented. This framework was specifically designed for URA applications but may find applications in other fields as well. An enhanced decoding algorithm was proposed for this framework that has the potential to simultaneously improve performance and decrease computational complexity. This enhanced decoding algorithm was applied to two URA schemes: coded compressed sensing (CCS) and CCS for massive MIMO. In both cases, PUPE performance gains were observed, and decoding complexity was significantly reduced.

The proposed algorithm is a natural extension of the existing literature. From coding theory, we know that there are at least three methods for inner and outer codes to interact. Namely, the two codes may operate completely independent of one another in a Forney-style concatenated fashion; this is the style of the original CCS decoder presented in [5]. Secondly, information messages may be passed between inner and outer decoders as both decoders converge to the correct codeword; this is the style of CCS-AMP, which was proposed by Amalladinne et al. in [7]. Finally, a successive cancellation decoder may be employed in the spirit of coded decision feedback; this is the style highlighted in this article and considered in [28,29]. Thus, the dynamic pruning introduced in this paper can be framed as an application of coding theoretic ideas to a concatenated coding structure that is common within URA.

By using the examples presented in this article pertained to CCS, we emphasize that dynamic pruning may be applicable to many algorithms beyond CCS. For instance, this approach may be relevant to support recovery in exceedingly large dimensions, where a divide-and-conquer approach is needed. As long as the inner and outer codes subscribe to the structure described in Section 2, this algorithmic enhancement can be leveraged to obtain performance and/or complexity improvements.

## Data Availability

No data to report.
